# Book Review: “Integrated Procedures in Facial Cosmetic Surgery” ISBN 978-3-030-46992-4 ISBN 978-3-030-46993-1 (eBook) © Springer Nature Switzerland AG 2021

**DOI:** 10.1186/s40902-022-00333-x

**Published:** 2022-01-29

**Authors:** Tae-Geon Kwon

**Affiliations:** Department of Oral & Maxillofacial Surgery, School of Dentistry, Kyungpook National University, Samduck 2 Ga, Jung Gu, Daegu, 700-421 Korea

**Fig. 1:**
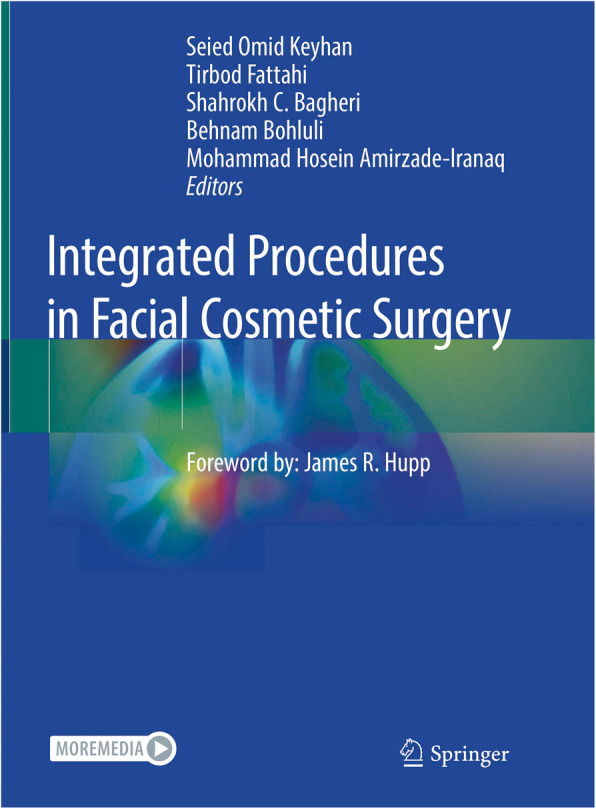
Cover of textbook

This book covers basics for facial cosmetic surgery, hard and soft tissue surgical technique, current advancements in cleft and orthognathic surgeries, and various updates of technical and principles for facial cosmetic surgery (Fig. 1). From the introduction, the book well demonstrate historical background of facial cosmetic surgery, which is useful to understand the basis of current techniques. It is informative to read this book because the contents are written based on the professional experiences from 26 well-known surgeons’ expertise.

Various field of facial cosmetic surgeries are fully discussed and contemporary surgical techniques are explained with readily visible illustrations and photographs. At the same time, four chapters are accompanied with videos that is quite useful for clinicians. Interestingly, the book contains lifting procedures as an important part of the cosmetic surgery, which reflects current needs and trends of cosmetic facial procedures. This book also contains the office-based procedures such as filler, botulinum toxin, laser, hair restoration, and various non-surgical rejuvenation procedures. It is quite interesting to note that at the end of each part arch as facial contouring (Part III & Part V), rhinoplasty (Part VI), office-based procedures (Part VII), blepharoplasty (Part IX), lifting procedures (Part X), orthognathic surgery (Part XI), and intra-oral plastic surgeries (Part XII), the Q & A discussion or commentaries are accompanied. These sections enables in-depth explanation of a techniques and highlight the important points of the surgical techniques. At the same time, the editors tried to give a chance to think about the critical aspects of each surgical principles to the readers.

This comprehensive text composed of 73 chapters provides practical guides for facial cosmetic surgery for not only of the training doctors but also for the specialists. The authors of the book tried to reflect the current enthusiasm and demands of clinical practitioners. And the texts are complemented by tables, figures, and various schematic illustrations; anatomy, surgical technique, and surgical concepts. This is an excellent resource and reference for surgeons tries to perform facial cosmetic procedures as well as for specialists who want to expand the knowledge and skills of cosmetic surgery. This book fully covers the current advanced techniques in cosmetic surgery and would be able to contribute the advancement of this surgical field.

